# Endorepellin remodels the endothelial transcriptome toward a pro-autophagic and pro-mitophagic gene signature

**DOI:** 10.1074/jbc.RA118.002934

**Published:** 2018-06-19

**Authors:** Thomas Neill, Eva Andreuzzi, Zi-Xuan Wang, Stephen C. Peiper, Maurizo Mongiat, Renato V. Iozzo

**Affiliations:** From the ‡ Department of Pathology, Anatomy, and Cell Biology, and the Cancer Cell Biology and Signaling Program, Sidney Kimmel Medical College at Thomas Jefferson University, Philadelphia, Pennsylvania 19107 and; the § Department of Translational Research, Experimental Oncology Division 2, CRO Aviano-IRCCS, National Cancer Institute, Aviano 33081, Italy

**Keywords:** parkin, mitochondria, mitochondrial membrane potential, vascular endothelial growth factor (VEGF), basement membrane, mitochondrial depolarization, mitofusin 2, mitostatin, Parkin, VEGFR2

## Abstract

Regulation of autophagy by proteolytically cleaved fragments of heparan sulfate proteoglycans is a novel and current research focus in tumor biology. Endorepellin is the C-terminal angiostatic fragment of the heparan sulfate proteoglycan perlecan and induces autophagy in endothelial cells. To further investigate this property, we used NanoString, a digital PCR platform for measuring pre-defined transcripts in biological samples to analyze a custom subset of 95 autophagy-related genes in human umbilical vein endothelial cells treated with ultrapure human recombinant endorepellin. We discovered an endorepellin-evoked pro-autophagic and pro-mitophagic gene expression signatures, which included two coordinately up-regulated mitochondrial-associated genes encoding the E3 ubiquitin protein ligase Parkin and the tumor suppressor mitostatin. Induction of both proteins required the tyrosine kinase activity of vascular endothelial growth factor receptor 2 (VEGFR2). Furthermore, we discovered that endorepellin evoked mitochondrial depolarization in endothelial cells via a specific interaction between its two proximal LG1/2 domains and VEGFR2. We also found that following loss of membrane potential, mitostatin and parkin interact and that mitostatin associates with the established Parkin receptor mitofusin-2. In conclusion, we have identified a critical role for endorepellin in remodeling the autophagic transcriptome and influencing mitochondrial homeostasis.

## Introduction

Autophagic regulation by proteolytically cleaved fragments of heparan sulfate proteoglycans (HSPGs)^2^ represent a novel thread of research in tumor biology (1–6) and may represent viable and novel therapeutic opportunities (7) outside conventional roles of the heparan sulfate chains (8–11). Perlecan, which encompass one of the largest multimodular HSPGs found in vascular basement membranes (12–18) and the osteocyte pericellular matrix (19), exhibits angiogenic bivalency by fine-tuning pro- and anti-angiogenic signals (20–22). Endorepellin, the C-terminal domain of perlecan, exhibits potent angiostatic properties (23) and is antithetic in function to the N-terminal HS chains required for growth factor sequestration and co-receptor activity (24, 25). Endorepellin is proteolytically liberated from perlecan *in vivo* (27) by the diverse family of matrix metalloproteinases (28–34). As an example of angiogenic fine tuning, matrix metalloprotease 9 cleaves angiostatic multimerin 2 for vessel sprouting (35).

Structurally, endorepellin is composed of three laminin G– like domains (LG1/3), each separated by twin EGF-like modules (2). Specifically, the modules separating LG2 from LG3 are sensitive to proteolysis and are cleaved by BMP1/Tolloid-like proteases to release the LG3 domain (36). Furthermore, the crystal structure of the C-terminal LG3 domain of endorepellin has been solved (37) and is emerging as an easily accessible biomarker (38) for an ever increasing array of diseases (39–48) including breast cancer (49).

Soluble endorepellin potently “repels” endothelial cell movements, thereby compromising cell migration and capillary morphogenesis (23). At the level of ligand/receptor interactions, endorepellin engages in a “dual receptor antagonism” (50) as a molecular tether, simultaneously ligating VEGFR2 and the α2β1 integrin (50). The molecular architecture of this heterotrimeric complex has been elucidated (50, 51). The proximal LG1/2 domains interact with the VEGFR2 ectodomain in a region spanning IgG repeats 3–5 (51, 52). In contrast, the terminal LG3 domain interacts with the α-I domain of the α2 subunit of the α2β1 integrin (50), thereby completing the bridge. This complex binding paradigm underscores the exquisite specificity (50) and sensitivity (53) of endorepellin toward endothelial cells. Downstream of dual receptor antagonism, endorepellin attenuates multiple signaling pathways conducive to a pro-angiogenic program via internalization of the receptor complex (4). Concurrent with the inhibition of the phosphatidylinositol 3-kinase/Akt/mammalian target of rapamycin and activation of AMP-activated protein kinase α (53, 54), we found that endorepellin evokes endothelial cell autophagy downstream of VEGFR2, a process concomitantly required for angiostasis (55). During autophagy, endorepellin transcriptionally up-regulates key pro-autophagic genes including *PEG3*, *BECN1*, and *MAP1LC3A* (53). However, a comprehensive understanding of the autophagic transcriptome engaged by endorepellin remains ill-defined.

In this study, we generated a custom NanoString probe-set encompassing 95 autophagy-related genes and probed mRNA of endothelial cells exposed for 6 h to soluble endorepellin. We found a unique endorepellin transcriptomic signature consisting of 23 differentially modulated genes. Of these, two genes, *TCHP* (mitostatin) and *PARK2* (Parkin), were involved in mitochondrial dynamics and mitophagy. We discovered that endorepellin stabilized the PINK1/Parkin quality control system following mitochondrial depolarization downstream of VEGFR2. Moreover, we observed that endorepellin evoked co-localization and binding of mitostatin to Parkin. These findings reveal the depth of endorepellin-evoked endothelial cell autophagy via transcriptome remodeling and open possibilities for extracellular matrix communication to the mitochondria in the regulation of angiostasis.

## Results

### Endorepellin differentially regulates the autophagic transcriptome

Mounting evidence indicates that stable autophagic programs, such as those influenced by endorepellin, require sustained transcriptional responses (56–58). Therefore, we designed a NanoString probe-set to measure the expression of 95 established autophagic genes induced by exposure to nanomolar concentrations of human recombinant endorepellin. NanoString is an innovative, state-of-the-art next generation digital PCR platform that is rapidly gaining attention in many diverse fields. It utilizes unique molecular barcodes to quantitate the number of user-defined transcripts present within each sample (59–63).

**Figure 1.**
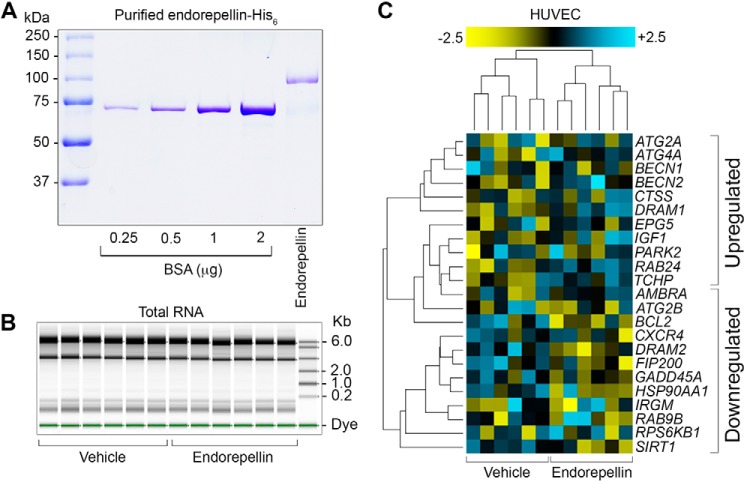
**Endorepellin differentially regulates the autophagic transcriptome.** *A,* SDS-PAGE demonstrating purity of human recombinant endorepellin. *B*, Agilent 2200 TapeStation-mediated analysis of RNA integrity and purity via capillary gel electrophoresis. *C*, hierarchical clustering of the 23 differentially modulated genes that were up-regulated (*n* = 11) or down-regulated (*n* = 12) in HUVEC in vehicle or endorepellin-treated samples. These 23 genes were filtered and selected on two criteria: >2-fold change (in either directions) and *p* < 0.05. Please refer to Tables S1 and S2 for further details. Data in *B* and *C* represent 6 independent biological replicates of either vehicle (PBS) or endorepellin (6 h, 200 nm)-treated early-passage (P3) HUVEC.

Early-passage (P3) human umbilical vein endothelial cells (HUVEC) were treated with highly purified human endorepellin produced in our laboratory (23) expressed in 293-EBNA cells as a C terminus His_6_-tagged protein (Fig. S1*A*) followed by nickel-nitrilotriacetic acid affinity purification. As determined by SDS-PAGE, our endorepellin preparations were free of any co-purifying contaminants (Fig. 1*A*). We administered endorepellin in full-serum (nutrient-rich) conditions and extracted total RNA from vehicle (sterile PBS) and endorepellin-treated HUVEC (*n* = 6/condition). RNA integrity was analyzed via an Agilent 2200 TapeStation and found to be ultrapure, intact, and suitable for NanoString transcriptomic analyses (Fig. 1*B*). We identified 23 differentially regulated genes that met our inclusion criteria, 2-fold induction or >50% suppression with a concurrent significance of *p* < 0.05 (Fig. 1*C*): 11 up-regulated (Table S1) and 12 down-regulated targets (Table S2). The pro-autophagic signature borne out by NanoString provided an unbiased and independent confirmation of endorepellin-evoked *BECN1* transcriptional induction (Fig. 1*C*) (53) with a simultaneous down-regulation of *BCL2*, a known autophagic inhibitor (Fig. 1*C*) (64, 65).

We focused on the coordinate up-regulation of *PARK2*, which encodes Parkin, and *TCHP*, which encodes mitostatin. Parkin is widely accepted as an RBR E3-ubquitin ligase commonly implicated in autosomal-recessive Parkinson's disease (66), and mitostatin is a poorly characterized tumor suppressor gene that we discovered as a decorin-inducible gene in cancer cells (67, 68). Importantly, both Parkin and mitostatin have been implicated in mitochondrial quality control and mitophagy (67, 69–73).

### Endorepellin co–up-regulates mitostatin and Parkin downstream of VEGFR2

We validated the selected NanoString targets by immunoblotting in two different, genetically normal endothelial cell types: HUVEC and Telo-HAEC. The latter are human aortic endothelial cells (HAEC) immortalized by the stable expression of telomerase (hereafter referred to as HAEC). These cell types are comparable in terms of possessing stable genomes, similar exposure to fluid shear stress, and responsiveness to nanomolar concentrations of endorepellin (50, 51, 54, 74).

**Figure 2.**
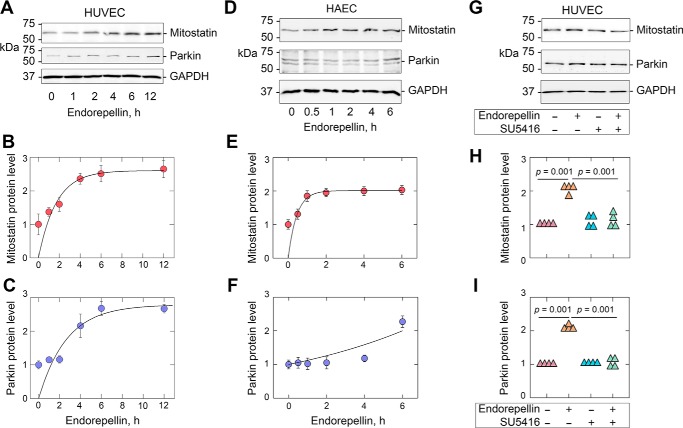
**Endorepellin co–up-regulates mitostatin and Parkin downstream of VEGFR2.** *A–C,* immunoblot and quantification of mitostatin and Parkin in HUVEC treated with endorepellin over time. *D–F*, identical experiments as in *A–C* in HAEC. *G–I*, immunoblot and quantification of mitostatin and Parkin in HUVEC treated in combination with endorepellin or SU5416 (30 μm) for 6 h. GAPDH served as an internal loading control for all immunoblots. Quantifications presented in *B, C, E, F, H,* and *I* are representative of at least three to four independent biological replicates in HUVEC or HAEC. Statistical analyses presented in *H* and *I* were calculated via one-way ANOVA.

We found that endorepellin induced mitostatin and Parkin over time (Fig. 2, *A–C*). In HUVEC, both proteins reached maximal levels at 6 h and remained elevated for up to 12 h. In HAEC, we observed similar kinetics for mitostatin that noticeably increased in as little as 1 h (Fig. 2*D*) and reached a plateau at ∼2 h (Fig. 2, *D* and *E*). In contrast, Parkin remained essentially unchanged until the terminal time point (6 h), where the magnitude of Parkin overlapped with that of mitostatin (Fig. 2, *D* and *F*).

As endorepellin requires both binding to VEGFR2 as well as its kinase activity to evoke angiostasis and autophagy (4, 21, 75), we next evaluated whether endorepellin would also require VEGFR2 signaling for Parkin and mitostatin up-regulation. We found that blocking the VEGFR2 tyrosine kinase with the selective and reversible ATP-competitive inhibitor, SU5416 (76, 77), significantly abrogated endorepellin-evoked Parkin and mitostatin induction (Fig. 2, *G–I*). Taken together, these results indicate a time-dependent increase in endorepellin-evoked Parkin and mitostatin, with differing kinetics, in HUVEC and HAEC downstream of VEGFR2.

### Endorepellin evokes mitochondrial depolarization via LG1/2

It is well established that Parkin is recruited to depolarized and/or damaged mitochondria (66, 79, 80). Thus, we hypothesized that endorepellin could compromise mitochondrial membrane potential integrity via Parkin modulation. We assessed the effects of endorepellin on mitochondrial membrane potential (ΔΨm) using JC-1, a lipophilic cationic dye that is sensitive to voltage fluctuations. JC-1 accumulates within the inner mitochondrial membrane in response to the ΔΨm. At a low ΔΨm (*e.g.* loss of membrane potential), JC-1 is monomeric and exhibits green fluorescence. However, high levels of JC-1 accumulate (proportional to a high ΔΨm) and lead to the formation of JC-1 aggregates that shifts the JC-1 emission spectrum toward red fluorescence (81). To this end, we treated HUVEC with endorepellin in parallel with carbonyl cyanide *m*-chlorophenylhydrazine (CCCP) or carbonyl cyanide *p*-trifluoromethoxyphenylhydrazone (FCCP), both known ionophores that uncouple ATP synthesis by transporting hydrogen ions across the mitochondrial membranes and dissipating the proton motive force.

**Figure 3.**
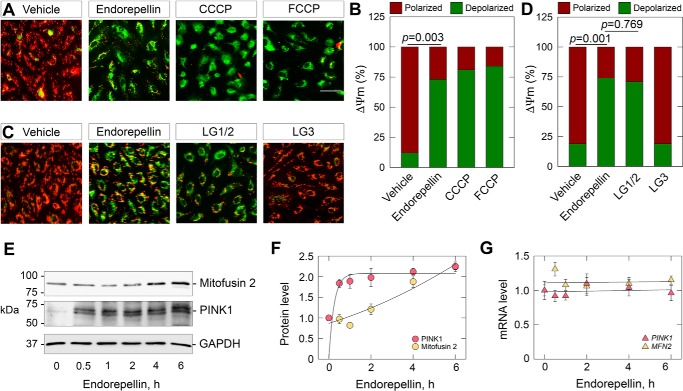
**LG1/2 domain of endorepellin is sufficient for mitochondrial depolarization.** *A* and *B,* representative fluorescence micrographs depicting live cell imaging of HUVEC after incubation with endorepellin (6 h), CCCP (1 h, 30 μm), or FCCP (10 min, 500 nm) in *A*, or endorepellin (6 h), LG1/2 (6 h, 150 nm), or LG3 (6 h, 150 nm) in *B*. HUVEC were cultured in nutrient-rich media and incubated with JC-1 (20 min, 7.5 μm) to assess mitochondrial membrane potential. *Scale bar* ∼80 μm. *C* and *D,* quantification of polarized (*green*) compared with depolarized (*red*) mitochondria as shown in *A* or *B*, respectively. *E* and *F*, immunoblot and quantification of mitofusin 2 and PINK1 in HUVEC treated with endorepellin over time. *G*, analyses of *PINK1* or *MFN2* in HUVEC treated with endorepellin over time. For live cell imaging in *A* and *B*, at least 10 fields per condition were acquired for each of three to four biological replicates in HUVEC. Quantifications in *C* and *D* are representative of three to four independent biological replicates. GAPDH served as an internal loading control for immunoblots in *E* and are representative of four independent biological replicates. Immunoblot quantifications in *F* are representative of four independent biological replicates. Gene expression analyses presented in *G* have been normalized to ACTB and represent four independent biological replicates. Statistical analyses presented in *C* and *D* were calculated via one-way ANOVA.

We found a profound loss of ΔΨm as determined by JC-1 staining (Fig. 3*A*) and further quantification of the green (depolarized, JC-1 monomers) and red (polarized, JC-1 aggregates) fluorescence (Fig. 3*B*). The magnitude of depolarization was not significantly different between endorepellin and CCCP (*p* = 0.652) or endorepellin and FCCP (*p* = 0.443), indicating comparable ΔΨm efficiency and a potential common pathway.

In an effort to identify the domains required for endorepellin binding to VEGFR2 (51), we generated two endorepellin fragments containing LG1/2 (composed of LG1, LG2, and both sets of the twin EGF-like modules) (Fig. S1*A*) or simply LG3 alone (Fig. S1*A*). We have previously shown that LG1/2 binds the IgG_3–5_ repeats of the VEGFR2 ectodomain, whereas LG3 binds the α2β1 integrin (4, 52, 75, 82). Thus, we employed these fragments to dissect which bioactive modules of endorepellin are responsible for transducing ΔΨm pertinent information. Notably, LG1/2 resulted in a robust ΔΨm akin to endorepellin (Fig. 3*C*). In contrast, LG3 was unable to evoke such a response (Fig. 3*C*). Quantifying the fluorescence intensity for both channels, we found a significant depolarization upon LG1/2 treatment (Fig. 3*D*) that was not statistically significantly different from endorepellin (*p* = 0.769), whereas LG3 was unchanged relative to control levels (*p* = 0.905). As ΔΨm was unchanged by LG3, we surmised blocking the α2β1 integrin with a specific mAb, mAb1998Z (53, 83), should have no apparent effect on endorepellin-evoked mitochondrial depolarization. Intriguingly, we found that endorepellin in the presence of mAb1998Z prevented ΔΨm when compared with endorepellin alone (Fig. S2, *A* and *D*). We validated endorepellin and mAb199Z with phallodoin staining to visualize the actin cytoskeleton. Endorepellin evokes actin dissolution via the α2β1 integrin (83), specifically LG3 (51, 82). Indeed, abrogating binding of endorepellin to the α2β1 integrin inhibited actin dissolution (Fig. S2*B*), thereby functionally validating mAb1998Z. Next, we combined LG1/2 with mAb1998Z and found loss of membrane potential independent of the α2β1 integrin blocking activities of the antibody (Fig. S2, *C* and *E*). Collectively, these data reinforce the concept that VEGFR2 is the primary receptor for endothelial cell ΔΨm, irrespective of the α2β1 integrin. These data indicate that LG1/2 is sufficient for mitochondrial depolarization and is most likely transduced by VEGFR2.

### Endorepellin-evoked modulation of PINK1 and mitofusin 2

Having established that endorepellin compromises ΔΨm, we next examined whether endorepellin would also affect PINK1, an effector kinase stabilized in response to mitochondrial depolarization (84), which works in synergy with Parkin (66). Among the targets of PINK1 is mitofusin 2 that acts as a receptor for recruited Parkin to cull damaged mitochondria (85). We found a rapid and sustained accumulation of PINK1 (within 30 min) following endorepellin treatments (Fig. 3, *E* and *F*). This was followed, at later time points, by a concomitant accumulation of mitofusin 2 protein (Fig. 3, *E* and *F*). We complemented these results in HUVEC with CCCP and FCCP and found comparable increases in both PINK1 over time with CCCP (Fig. S1*B*) and PINK1 transiently with FCCP (Fig. S1*C*) in HUVEC. Notably, these increases in PINK1 and mitofusin 2 occurred independently of transcription, as no modulation in the mRNA profiles of either *PINK1* or *MFN2* was found (Fig. 3*G*). Thus, we have identified a novel downstream signaling pathway for endorepellin that triggers mitochondrial depolarization via the LG1/2 domain with a consequent increase in PINK1 and mitofusin 2, independent of transcription.

### VEGFR2 is required for endorepellin-evoked mitochondrial depolarization

**Figure 4.**
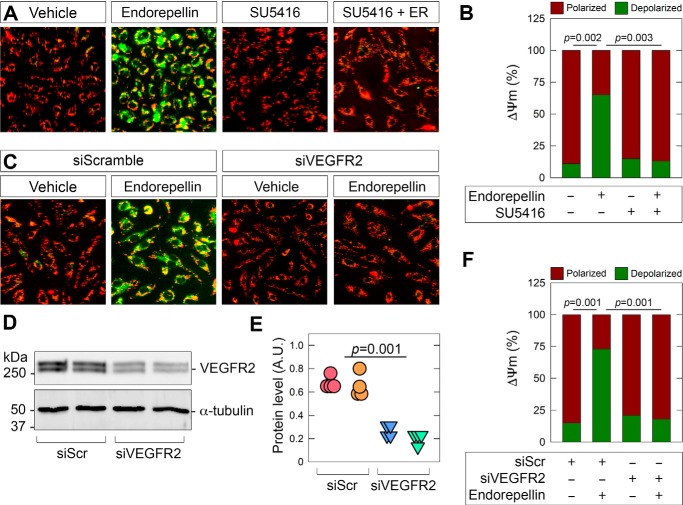
**VEGFR2 is required for endorepellin-evoked mitochondrial depolarization.** *A*, representative fluorescence micrographs depicting live cell imaging of HUVEC after incubation with endorepellin in combination with SU5416 after staining with JC-1. HUVEC were cultured in nutrient-rich media. *B*, quantification of polarized (*green*) compared with depolarized (*red*) mitochondria as shown in *A. C*, representative fluorescence micrographs depicting live cell imaging of HUVEC after transient transfection of scramble siRNA (siScramble) or siVEGFR2 followed by endorepellin (6 h). *D* and *E*, immunoblot and quantification of VEGFR2 silencing in HUVEC. *F*, quantification of polarized (*green*) compared with depolarized (*red*) mitochondria as shown in *C. Scale bar* in *A* and *C,* ∼80 μm. For live cell imaging in *A* and *C*, at least 10 fields per condition were acquired for each of four biological replicates in HUVEC. Quantifications in *B* and *F* are representative of four independent biological replicates. GAPDH served as an internal loading control for immunoblots in *D* and are representative of four independent biological replicates. Immunoblot quantifications in *E* are representative of four independent biological replicates. Statistical analyses presented in *B* and *F* were calculated via one-way ANOVA.

Having ascertained that LG1/2 is sufficient for mitochondrial depolarization, we evaluated whether the tyrosine kinase of VEGFR2 is required. Using JC-1 to assess the effects of endorepellin on ΔΨm, we found that blocking VEGFR2 kinase with SU5416 significantly blocked endorepellin-evoked ΔΨm (Fig. 4, *A* and *B*). Furthermore, treatment with SU5416 alone had no effect, suggesting an active process mediated exclusively upon endorepellin-binding VEGFR2 (Fig. 4, *A* and *B*). Next, we utilized siRNA to transiently deplete VEGFR2. Transient transfection of a scramble siRNA sequence complexed with a lipid-based carrier did not cause significant damage or undue stress (compare Fig. 4, *A* with *C*). After validating VEGFR2 depletion with a pool of 3–5 targeting siRNA oligonucleotides (Fig. 4*D*) with a resulting knockdown of ∼65% (Fig. 4*E*), we found that loss of the receptor recapitulated the effect of pharmacological inhibition with SU5416 (Fig. 4*C*) and significantly attenuated ΔΨm (Fig. 4*F*). Thus, we have identified a functional and mechanistic role for VEGFR2 in transducing information from endorepellin for mitochondrial depolarization in endothelial cells.

### Parkin and mitostatin colocalize in a VEGFR2-dependent manner

Next, we utilized confocal laser microscopy to determine whether there is any co-localization of Parkin, and mitostatin, and whether mitostatin and Parkin interact in solution. Moreover, mitostatin binds mitofusin 2 at specialized regions known as mitochondrial associated membranes juxtapositions between the mitochondria and endoplasmic reticulum (86).

**Figure 5.**
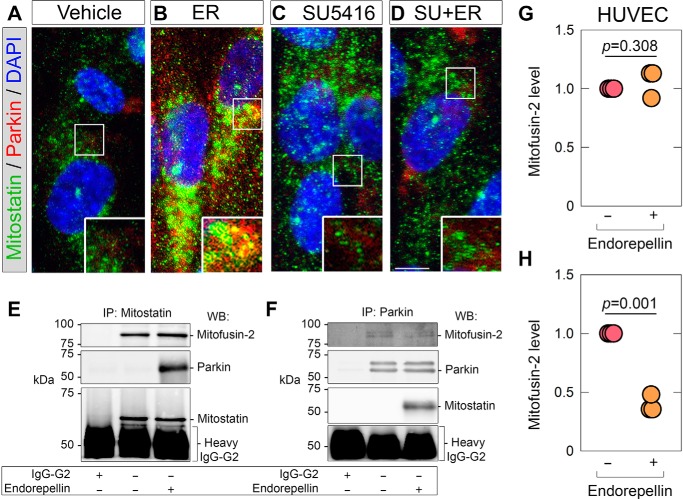
**Parkin and mitostatin co-localize in a VEGFR2-dependent manner.** *A–D,* confocal laser microscopy depicting mitostatin (*green*) and Parkin (*red*) following endorepellin (6 h) alone or in combination with SU5416 in HUVEC. Nuclei (*blue*) were visualized with 4′,6-diamidino-2-phenylindole (*DAPI*). *Scale bar* ∼10 mm. *Insets* denote 3 times digitally magnified images from the area demarcated by the *solid white box. E* and *F*, representative images of co-immunoprecipitations of HUVEC treated with endorepellin and immunoprecipitated for mitostatin (*E*) or Parkin (*F*) and immunoblotted for mitofusin 2, Parkin, or mitostatin. *G* and *H*, quantification of mitofusin-2 following mitostatin immunoprecipitation (as in *E*) or Parkin immunoprecipitation (as in *F*). For confocal microscopy in *A-D*, at least five fields/condition were obtained for at least two independent biological replicates in HUVEC. Immunoblots in *E* and *F* are representative of three independent biological replicates in HUVEC. Mitofusin-2 quantifications in *G* and *H* are representative of three independent biological replicates in HUVEC. Statistical analyses provided by two-tailed Student's *t* test.

Under basal conditions, we detected very little Parkin staining (Fig. 5*A*) in agreement with the biochemical data showing low levels detected by immunoblot (*cf*. Fig. 2*A*). Mitostatin immunoreactivity was considerably more robust with a profuse staining pattern throughout the cell and nuclear compartments (Fig. 5*A*). We found no co-localization of mitostatin and Parkin under resting conditions. Exogenous endorepellin caused a marked increase in Parkin throughout the cytosol and a striking co-localization of mitostatin and Parkin (Fig. 5*B*) in clearly delineated punctate structures (Fig. 5*B*, *inset*). As seen above with the ΔΨm assays, SU5416 alone had no effect on the distribution of mitostatin and Parkin (Fig. 5*C*). However, SU5416 completely blocked the effect of endorepellin on mitostatin and Parkin co-localization (Fig. 5*D*). These data suggest that VEGFR2 controls the association of mitostatin and Parkin in response to endorepellin.

Next, we corroborated our imaging study with immunoprecipitation data. Using the anti-mitostatin antibody, we successfully immunoprecipitated mitostatin. Lysates exposed to a control rabbit IgG-G2 displayed no such immunoprecipitates (Fig. 5*E*), validating the specificity of our antibody. Importantly, we found an exclusive interaction of mitostatin with Parkin following endorepellin treatment (Fig. 5*E*), thereby confirming our confocal microscopy results. We also confirmed the previously reported (86) basal association of mitostatin with mitofusin 2 (Fig. 5*E*) but found no altered interactions upon endorepellin treatment (Fig. 5*E*).

Next, we performed reciprocal co-immunoprecipitations of Parkin (Fig. 5*F*). Using a control mouse IgG-G2, we found no immunoprecipitating bands, substantiating the specificity of our antibody. In agreement with the immunofluorescence data, we found an exclusive association of Parkin with mitostatin following endorepellin treatment (Fig. 5*F*). However, immunoblotting for mitofusin 2 revealed a modest decrease in the association of Parkin and mitofusin 2 not seen when mitostatin was immunoprecipitated (Fig. 5*F*). These data suggest a differential binding of mitostatin and mitofusin 2 over Parkin in endothelial cells. Quantification of mitofusin-2 following immunoprecipitation of mitostatin (Fig. 5*G*) or Parkin (Fig. 5*H*) further confirmed these results.

Collectively, our results identify an exclusive association between mitostatin and Parkin after exposure to endorepellin. Furthermore, the formation of the mitostatin-Parkin complexes require the physical presence of VEGFR2 and the activity of its kinase.

### Endorepellin evokes autophagic flux, but not of mitofusin 2 or mitostatin

**Figure 6.**
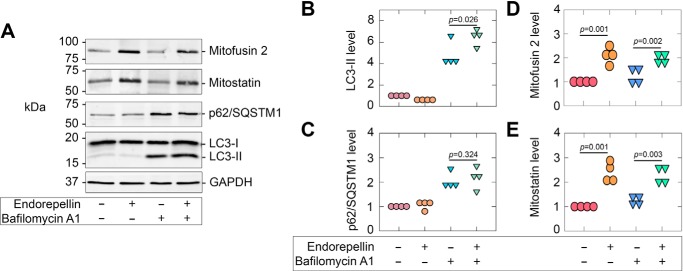
**Endorepellin evokes autophagic flux, but not of mitofusin 2 or mitostatin.** *A*, representative immunoblots of mitofusin 2, mitostatin, p62/SQSTM1, LC3-I/-II, and GAPDH following endorepellin (6 h) alone or in combination with bafilomycin A1 (100 nm). *B–E*, quantification of targets as shown in *A.* GAPDH served as an internal loading control for immunoblots in *A* and are representative of four independent biological replicates. Immunoblot quantifications in *B-E* are representative of four independent biological replicates. Statistical analyses presented in *B* and *C* were calculated via one-way ANOVA.

We have determined that endorepellin is capable of increasing the rate of endothelial cell autophagy (55) by examining autophagic flux of established markers (such as LC3-II) (87) in the presence of chloroquine. Here, we evaluated the effects of bafilomycin A1, a V-type ATPase inhibitor that prevents fusion between the autophagosome and lysosome, in conjunction with endorepellin thereby permitting analyses of the rates of accumulated autophagic markers. Treatment with bafilomycin A1 (100 nm) alone resulted in a marked accumulation of LC3-II (Fig. 6, *A* and *B*) and p62/SQSTM1 (Fig. 6, *A* and *C*). Intriguingly, under basal conditions at two different concentrations, bafilomycin A1 had no discernible effects on mitofusin 2 (Fig. 6, *A* and *D*) or mitostatin (Fig. 6, *A* and *E*).

In congruence with our previous study (55), endorepellin, in combination with bafilomycin A1, significantly enhanced autophagic flux as per LC3-II analysis (Fig. 6, *A* and *B*). However, under the same conditions, we found no significant increase (or change) in the levels of p62/SQSTM1 (Fig. 6, *A* and *C*). Likewise, as per the aforementioned data (*cf.* Figs. 2 and 3*E*), endorepellin evoked mitofusin 2 (Fig. 6, *A* and *D*) and mitostatin (Fig. 6, *A* and *E*). Intriguingly, a combination treatment of endorepellin and bafilomycin A1 increased neither mitofusin 2 (Fig. 6, *A* and *D*) nor mitostatin (Fig. 6, *A* and *E*) above that of simply endorepellin alone. Parkin responded in a similar manner (data not shown).

In summary, we recapitulated endorepellin-enhanced autophagic flux in endothelial cells. However, the newly identified targets were not sensitive to basal autophagic flux nor augmented flux as stimulated by endorepellin.

## Discussion

We discovered a unique endorepellin-evoked autophagic signature following digital PCR profiling of a specific subset of the endothelial cell transcriptome. Querying our custom probe-set, we found 23 differentially regulated genes. Among the targets, several genes including the distinct regulation of *BECN1* and the autophagic inhibitor *BCL2* (53, 64, 65, 88, 89) independently confirmed the robustness of our dataset and signals a shift to a pro-autophagic program. Beclin-1 is phosphorylated by Akt leading to autophagic inhibition (90). We discovered that endorepellin inactivates the phosphatidylinositol 3-kinase/Akt pathway (54) as a potential mechanism of Beclin 1 derepression and resultant autophagic initiation. Beclin 1 appears to be governed by RTKs (91), designating a convergence on RTKs by soluble matrix components in evoking autophagy (92). The Beclin-1 homologue *BECN2*, also implicated in autophagy and metabolism (93), was further identified as an endorepellin-inducible gene.

Moving forward, endorepellin co–up-regulates the mRNA and protein of *PARK2* and *TCHP* (encoding Parkin and mitostatin, respectively), effectors implicated in mitochondrial homeostasis and mitophagy (66, 73). Induction of Parkin and mitostatin depended on the VEGFR2 tyrosine kinase, indicating partial agonism, thereby allowing us to refine the model as a “dual receptor partial agonism” rather than strict dual receptor antagonism.

Given that Parkin is recruited to depolarized and/or damaged mitochondria for Parkin-mediated mitophagy (94), we discovered that endorepellin-evoked mitochondrial depolarization. Intriguingly, depolarization occurred with the same magnitude as CCCP and FCCP, known electron transport chain uncouplers. Functionally, and mechanistically, the modular architecture of endorepellin permitted us to identify the LG1/2/VEGFR2 interaction, but not the LG3/α2β1 interaction, as sufficient for the loss of mitochondrial membrane potential. Intriguingly, a combination of mAb1998Z with endorepellin significantly blocked ΔΨm. This might be due to a positioning role of the α2β1 integrin for full-length endorepellin to interact with VEGFR2. This is reinforced by LG1/2 in conjunction with mAb1998Z efficiently depolarizing the mitochondria and lack of response of LG3 alone. Alternately, endorepellin may have been blocked due to steric hindrance from the antibody binding to the αI-domain of the α2 subunit and unabridged endorepellin.

Whether mitochondrial depolarization occurs downstream of VEGFR2 in response to intracellular calcium mobilization (95, 96) remains unknown. However, based on our previous work (54) this mode of action seems unlikely. Endorepellin prevents phosphorylation of VEGFR2 at Tyr^1175^, a key phospho-site for phospholipase C-γ recruitment; thereby, precluding the formation of diacylglycerol and inositol 1,4,5-triphosphate. The latter being critical for calcium flux downstream of RTK signaling. Concurrent with mitochondrial depolarization was a rapid stabilization of PINK1 (94) and temporal accumulation of mitofusin 2, a mitostatin binding partner and Parkin receptor (85, 86, 97). Increased amounts of mitofusin 2 act to juxtapose Parkin near PINK1 for subsequent activation (97) and clearance of the damaged organelle.

The co-localized mitostatin/Parkin positive puncta formed in response to endorepellin, downstream of VEGFR2 signaling, was decidedly more discreet in morphology than autophagosomes evoked by endorepellin (53, 55). This interaction appeared to form only after stimulation, as no co-localization or binding was found under basal conditions. Using the previously published interaction between mitostatin and mitofusin 2 (86) as a positive control for our immunoprecipitation, we found an increased association between mitostatin and mitofusin 2 post-endorepellin treatment. Resolving the precise interactions and deciphering the biological consequence of the mitostatin/Parkin interaction and increased binding of mitostatin to mitofusin 2 will require future studies as it pertains to mitochondrial homeostasis.

The observation that Parkin, mitostatin, and mitofusin 2 are not substrates of basal autophagic flux or endorepellin-evoked flux in endothelial cells implies their function in an alternate pathway. Indeed, mitofusin 2, despite being involved in mitophagy, is sent to the 26S proteasome for degradation by Parkin (70). Emerging evidence posits a cooperation between the autophagic machinery and the ubiquitin-proteasome system for competent mitochondrial clearance via mitophagy (98). Considering that mitostatin-Parkin and mitostatin-mitofusin 2 are complexed, these protein complexes may thereby bypass the autophagosomal system and are instead being targeted for degradation.

In conclusion, we discovered a unique endorepellin-dependent transcriptomic signature revealing a broad remodeling of genes to favor the autophagic response. Furthermore, for the first time, we found evidence of endorepellin regulating mitochondrial dynamics. These findings have widened our understanding of matrix-driven autophagy and provide a framework conducive for a deeper understanding of this conserved process.

## Experimental procedures

### Cells, chemicals, and reagents

HUVEC were obtained from Lifeline Cell Technology (Frederick, MD), grown in basal medium and supplemented with the VascuLife EnGS LifeFactors Kit (Lifeline Cell Technology). HUVEC were used within the first five passages. Telo-HAEC were obtained from American Type Cell Cultures (Manassas, VA) and cultured in EGM-2 SingleQuot Kit Supplements and Growth Factors (Lonza). Rabbit polyclonal antibodies against GAPDH, PINK1, VEGFR2, and p62/SQSTM1 were obtained from Cell Signaling Technology. Rabbit polyclonal anti-LC3B, SU5416, CCCP, FCCP, and JC-1 were purchased from Sigma. The horseradish peroxidase-conjugated goat anti-rabbit, donkey anti-mouse secondary antibodies, and negative rabbit and mouse IgG-G2 immunoprecipitation antibodies were obtained from EMD Millipore (Billerica, MA). Mouse monoclonal antibodies against Parkin and mitofusin 2 were obtained from Cell Signaling Technology. Mouse mAb against α-tubulin was purchased from Santa Cruz Biotechnology. A custom rabbit polyclonal antibody against mitostatin was generated as described elsewhere (73). All primary antibodies were used at 1:1000 dilution in 1% BSA/TBST, except for GAPDH, which was used at 1:10,000 and α-tubulin, which was used at 1:400. For immunofluorescence, primary antibodies were used at 1:200 in 1% BSA in PBS. Secondary antibodies for chemiluminescence were used at 1:5,000 in the same buffer as above. SuperSignal West Pico enhanced chemiluminescence substrate was purchased from Thermo Fisher Scientific. The purification and validation of human recombinant endorepellin have been described and demonstrated above. Purification and validation of human recombinant LG1/2 and LG3 are described elsewhere (51). Highly purified endorepellin was used at 200 nm, whereas LG1/2 and LG3 was used at 150 nm throughout the study. Original, whole blots can be found in Fig. S3.

### NanoString transcriptomic analysis

NanoString nCounter reporter probe-sets were designed to detect expression changes in a manually curated set (*n* = 95) of established autophagy genes available in the literature at the time of probe-set synthesis.

Early passage HUVEC (P3) were treated (*n* = 6) with vehicle (PBS) or endorepellin (6 h, 200 nm). RNA was extracted and validated by an Agilent 2200 TapeStation before entering the workflow for NanoString analyses. Samples were processed (including RNA validation) at the Genomic Pathology Laboratory (Thomas Jefferson University), following the nCounter Gene Expression protocol. Briefly, total RNA was incubated at 65 °C with reporter and capture probes in hybridization buffer. Capture probes were purified and analyzed on the nCounter Digital Analyzer. The number of molecules of a given transcript in the endorepellin-treated samples was determined by normalizing detected transcript counts to the geometric mean of control RNA sequences and a set of control genes (*n* = 5) that did not show evidence of altered expression by endorepellin. Significant differences between vehicle and endorepellin (*p* < 0.05) were detected by paired two-tailed *t* tests comparing the paired mean values for each gene (averaged across samples within each condition) between each condition.

### Transient RNAi-mediated silencing

HUVEC were transiently transfected using Lipofectamine RNAiMAX (Life Technologies) mixed with siRNA against *Homo sapiens VEGFR2* (Santa Cruz Biotechnology). Scrambled siRNA (Santa Cruz Biotechnology) served as a control for all siRNA experiments presented herein. The protocol for siRNA-mediated silencing is described elsewhere (99).

### Mitochondrial membrane potential

At least three individual assays were performed in HUVEC using the mitochondrial dye JC-1. HUVEC were grown in four chambered glass slides coated with 0.1% gelatin for 24 h at 37 °C. Cells were treated as per the experimental conditions described herein. CCCP was used at 30 μm and FCCP used at 500 nm for the last hour or 10 min before staining with JC-1, respectively. Each chamber was incubated with JC-1 (7.5 μm) for 20 min. Cells were washed three times with PBS and imaged live using a Leica DM5500B microscope. All the images were procured using the same exposure, gain, and intensity.

### Immunofluorescence and confocal laser microscopy

Typically, ∼5 × 10^4^ HUVEC were plated on 0.2% gelatin-coated 4-well chamber slides (Nunc, Thermo Scientific) and grown to full confluence in their growth media at 37 °C. Cells were treated as per the experimental conditions contained herein, and immunofluorescence was performed as previously done (100, 101). Slides were incubated with conjugated secondary antibodies such as: goat anti-rabbit IgG Alexa Fluor® 488 and goat anti-mouse IgG Alexa Fluor® 564 (Invitrogen). Nuclei were visualized with 4′,6-diamidino-2-phenylindole (Vector Laboratories). JC-1 live cell immunofluorescence images were acquired with a ×10 objective on a LEICA DM5500B microscope installed with the Leica Application suite, using advanced fluorescence version 1.8 software (Leica Microsystems, Frankfurt, Germany). Confocal analyses were carried out utilizing a ×63, 1.3 oil-immersion objective of a Zeiss LSM-780 confocal laser-scanning microscope with Zen Imaging Software. A full description of the immunofluorescence and confocal laser microscopy protocol can be found elsewhere (78).

### Quantitative real-time PCR

Expression analysis by quantitative real-time PCR was carried out on subconfluent six-well plates seeded with ∼2 × 10^5^ of HUVEC and harvested in TRIzol reagent (Invitrogen) following the appropriate experimental conditions. Gene expression analysis was performed on a Roche LightCycler 480-II and calculated with the comparative *C_t_* method. A full description can be found in Ref. 26.

### Quantification and statistical analysis

Immunoblots were quantified by scanning densitometry using Scion ImageJ software (NIH). Graphs were generated using Sigma Stat 3.10. Experiments with three or more comparison groups were subjected to one-way ANOVA followed by a Bonferroni post hoc test. Differences among the conditions were considered significant at *p* < 0.05.

## Author contributions

T. N. and R. V. I. conceptualization; T. N. and R. V. I. data curation; T. N. and R. V. I. formal analysis; T. N. and R. V. I. writing-original draft; T. N. and R. V. I. writing-review and editing; E. A., Z.-X. W., S. C. P., and M. M. resources; Z.-X. W. and S. C. P. methodology; S. C. P. and R. V. I. funding acquisition.

## Supplementary Material

Supporting Information

## Footnotes

2 The abbreviations used are:

HSPG: heparan sulfate proteoglycan
CCCP: carbonyl cyanide *m*-chlorophenylhydrazine
FCCP: carbonyl cyanide *p*-trifluoromethoxyphenylhydrazone
HAEC: human aortic endothelial cells
HUVEC: human umbilical vein endothelial cells
LC3: microtubule associated light chain 3
LG: laminin G-like domains
PINK1: PTEN-induced putative kinase
p62/SQSTM1: sequestome 1
VEGFR2: vascular endothelial growth factor receptor 2
RTK: receptor tyrosine kinase
GAPDH: glyceraldehyde-3-phosphate dehydrogenase
ANOVA: analysis of variance
EGF: epidermal growth factor.
