# Supplementary material for: Endorepellin remodels the endothelial transcriptome toward a pro-autophagic and pro-mitophagic gene signature
Source: J Biol Chem. 2018 Jun 19;293(31):12137–48. doi: 10.1074/jbc.RA118.002934 (PMC6078466; doi:10.1074/jbc.RA118.002934)
Supplement: Supporting Information [file supp_293_31_12137__index.html]

Endorepellin remodels the endothelial transcriptome toward a pro-autophagic and pro-mitophagic gene signature — Endorepellin evokes a pro-autophagic gene signature — Supporting Information 

# Endorepellin remodels the endothelial transcriptome toward a pro-autophagic and pro-mitophagic gene signature

## Supporting Information

- Supporting Information - Three figures, 2 tables and additional refernces
