# Supplementary material for: Endorepellin remodels the endothelial transcriptome toward a pro-autophagic and pro-mitophagic gene signature
Source: J Biol Chem. 2018 Jun 19;293(31):12137–48. doi: 10.1074/jbc.RA118.002934 (PMC6078466; doi:10.1074/jbc.RA118.002934)
Supplement: Supporting Information [file supp_RA118.002934_136702_1_supp_156517_pjl0pf.pdf]

Supporting Information

Endorepellin remodels the endothelial transcriptome toward a pro-autophagic and pro-mitophagic gene signature

Neill et al

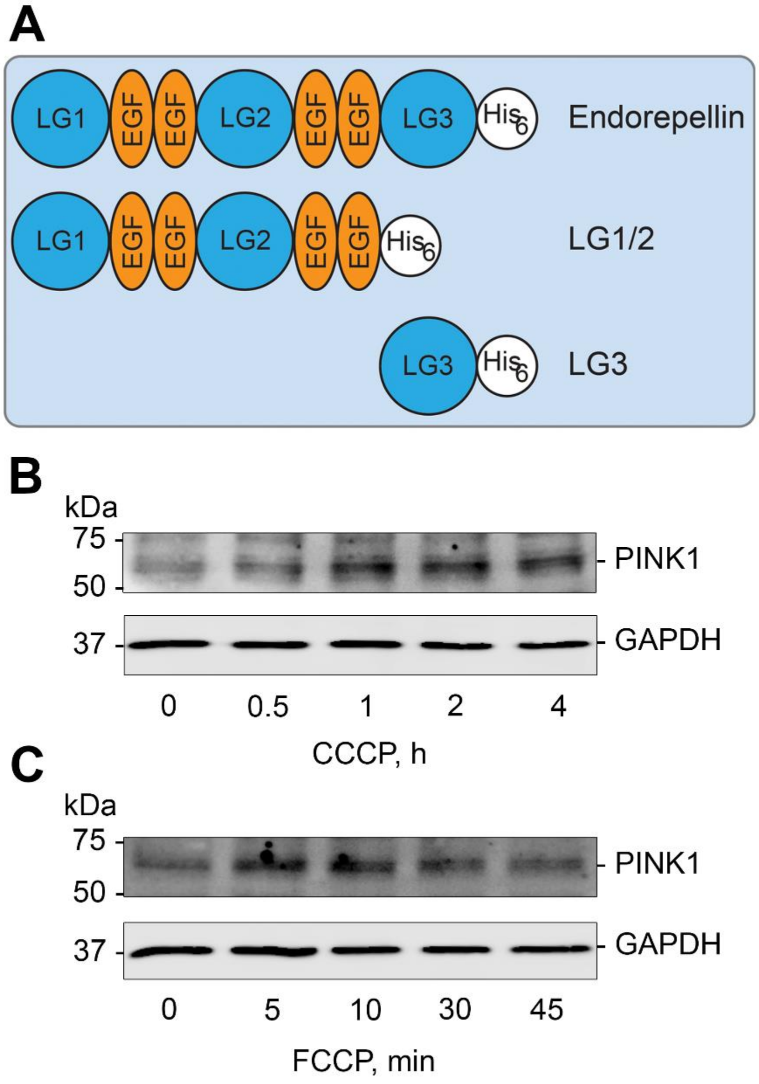

Figure S1. Endorepellin architecture and CCCP or FCCP stabilization of PINK1. *A*, schematic representation of His_6-tagged full-length human recombinant endorepellin and the serial truncations generated. *B,C*, representative immunoblot of PINK1 following CCCP *(B)* or FCCP *(C)* in HUVEC at the indicated time points. GAPDH served as a loading control in *B,C* are represents three independent biological replicates.

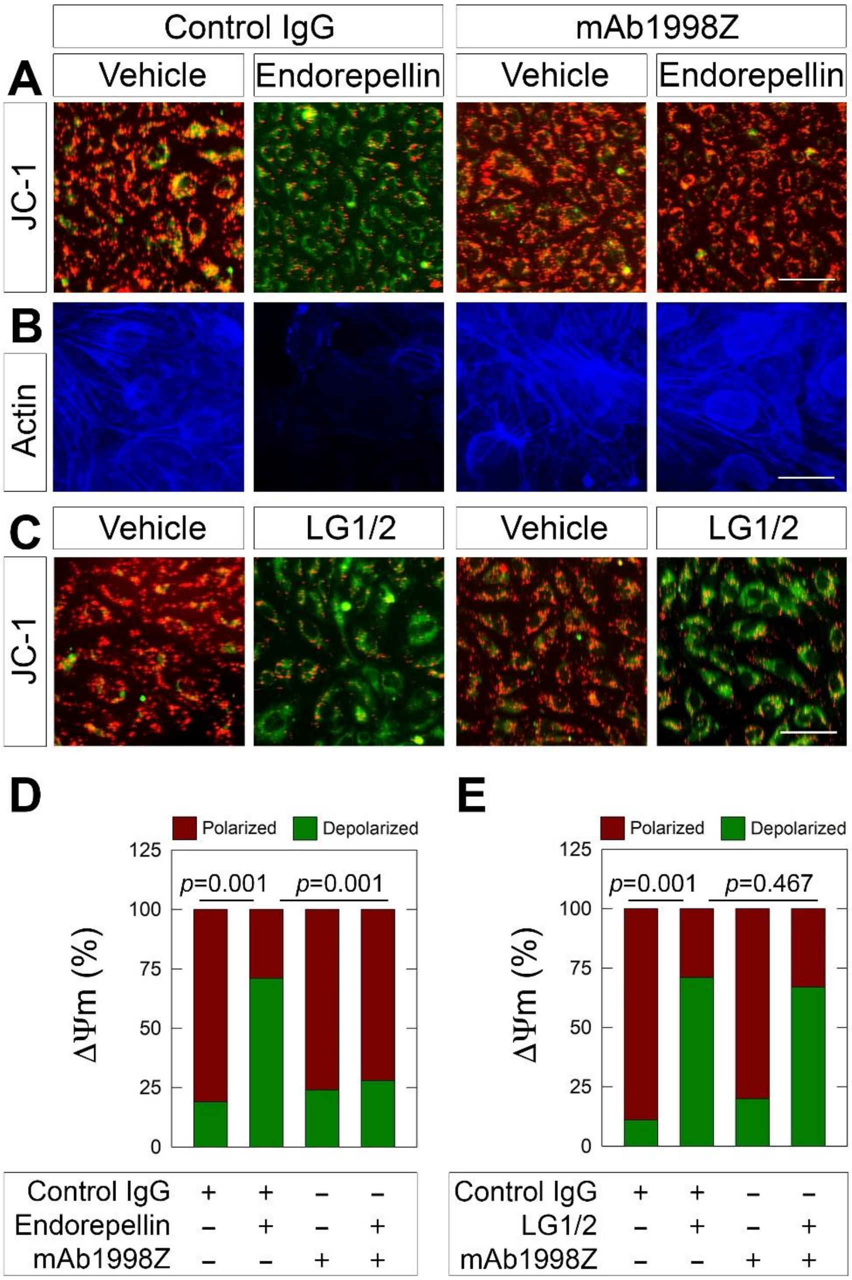

Figure S2. Mitochondrial depolarization proceeds independently of the α2β1 integrin. *A,B,* representative fluorescence micrographs depicting live cell imaging of HUVEC after incubation with endorepellin (6 h) in the presence of either a control IgG-G2 antibody (1 μg/ml) or endorepellin with mAb1998Z (1 μg/ml) and stained with JC-1 (*A*) or phalloidin (*B*). C, representative fluorescence micrographs depicting live cell imaging of HUVEC after incubation with LG1/2 (6 h) with either a control IgG-G2 antibody (1 μg/ml) or LG12 with mAb1998Z (1 μg/ml) and stained with JC-1. Scale bar ~80 μm in *A,C*; ~10 μm in *B*. *D,E,* quantification of polarized (green) compared with depolarized (red) mitochondria as shown in *(A)* or *(C)*, respectively. For live cell imaging in *(A)* and *(C),* at least 10 fields per condition were acquired for each of four biological replicates in HUVEC. Quantification in *D,E*, are representative of four independent biological replicates. Statistical analyses presented in (*D*) and (*E*) were calculated via one-way ANOVA.

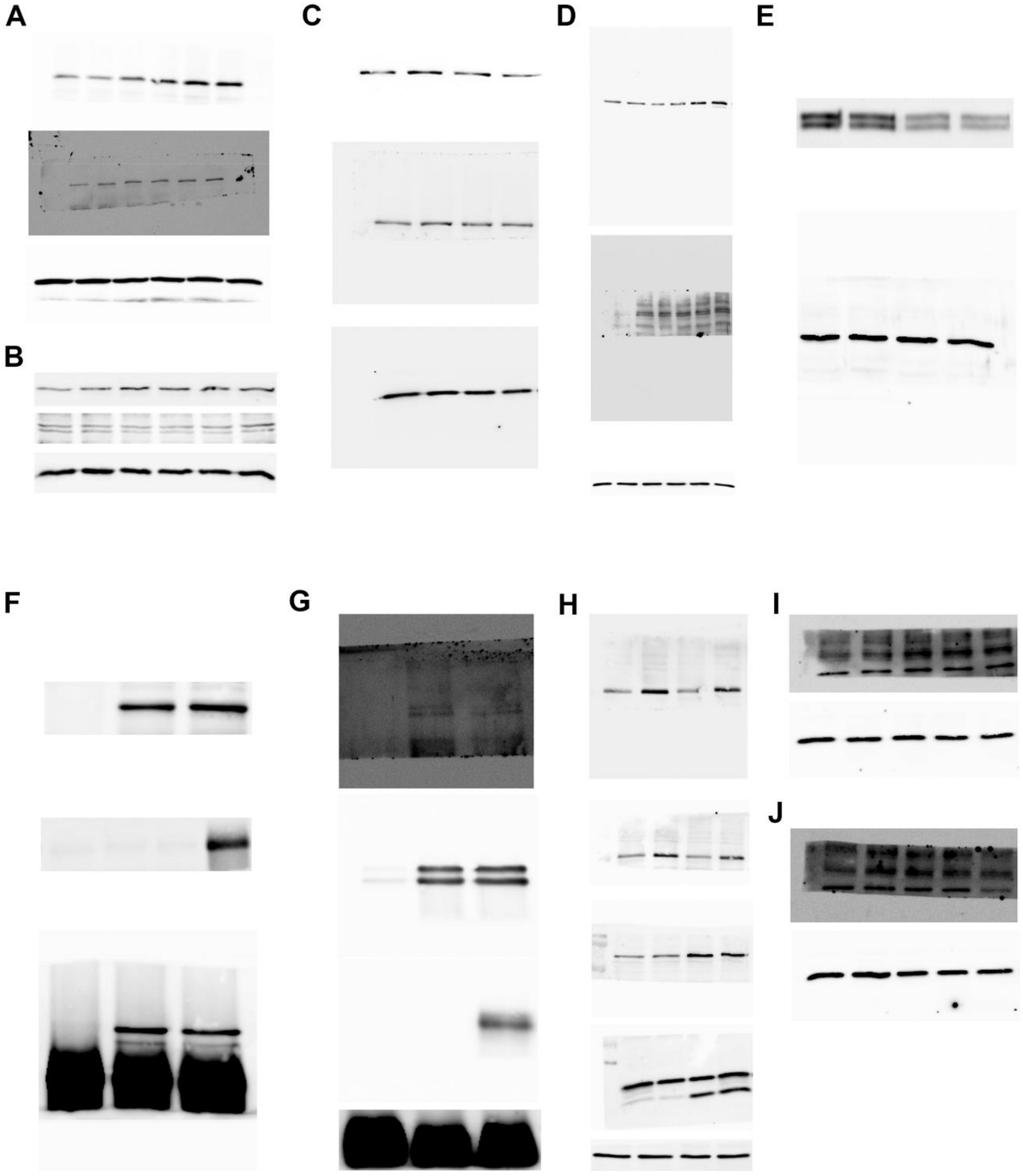

Figure S3. Gallery of original immunoblots. *A-C*, immunoblots used in Fig. 2*A,D,G*, respectively. *D*, immunoblots used in Fig. 3*E*. *E*, immunoblots used in Fig. 4*D*. *F,G*, immunoblots used in Fig. 5*E,G*, respectively. *H*, immunoblots used in Fig. 6*A*. *I,J*, immunoblots used in Fig. S1*B,C*, respectively. Immunoblots are presented here appear in the same sequence (e.g. from top to bottom) as in the figures for which they used.

Table S1. Significantly (2-fold induced with $p<0.05$) upregulated autophagy genes in early passage (P2) HUVEC following endorepellin (200 nM, 6h) treatment compared with vehicle (PBS) treated cells. Data are representative of six independent, biological replicates each for vehicle or endorepellin.

Symbol	Gene name	Fold	SEM (±)	*p*-value	References
ATG2A	Autophagy-related protein 2 homolog A	+2.36	0.67	*0.02*	(1,2)
ATG4A	Autophagy-related protein 4 homolog A	+2.47	0.17	*1.32 E-5*	(3)
BECN1	Beclin 1	+2.01	0.44	*0.03*	(4,5)
BECN2	Beclin 2	+3.63	1.15	*0.03*	(6)
CTSS	Cathepsin S	+2.31	0.51	*0.01*	(7)
DRAM1	DNA damage regulated autophagy modulator 1	+2.34	0.14	*4.49 E-6*	(8)
EPG5	Ectopic P-granules autophagy protein 5 homolog	+2.77	0.20	*9.65 E-6*	(9)
IGF1	Insulin like growth factor 1	+2.37	0.09	*8.63 E-9*	(10)
PARK2	Parkin RBR E3 ubiquitin protein ligase	+2.78	0.19	*6.15 E-6*	(11,12)
RAB24	Ras-associated protein 24	+2.35	0.09	*1.82 E-7*	(13)
TCHP	Trichoplein keratin filament binding / mitostatin	+2.71	0.31	*0.0003*	(14-17)

Table S2. Significantly (2-fold suppressed with *p*<0.05) downregulated autophagy genes in early passage (P2) HUVEC following endorepellin (200 nM, 6h) treatment compared with vehicle (PBS) treated cells. Data are representative of six independent, biological replicates each for vehicle or endorepellin.

**Symbol**	**Gene name**	**Fold**	**SEM (±)**	***p*-value**	**References**
AMBRA1	Autophagy and Beclin 1 regulator	-2.38	0.16	*9.09 E-9*	(18-21)
ATG2B	Autophagy-related protein 2 homolog B	-2.70	0.15	*8.77 E-12*	(2,22)
BCL2	B-cell CLL/lymphoma 2	-2.50	0.08	*6.94 E-13*	(23,24)
CXCR4	C-X-C motif chemokine receptor 4	-2.63	0.28	*7.36 E-8*	(25,26)
DRAM2	DNA damage regulated autophagy modulator 2	-2.56	0.18	*1.13 E-10*	(8)
FIP200	FAK family kinase-interacting protein of 200 kDA	-3.84	0.41	*4.21 E-10*	(27)
GADD45A	Growth arrest and DNA damage-inducible 45 alpha	-2.22	0.10	*8.55 E-10*	(28-30)
HSP90AA1	Heat shock protein 90 alpha family class A member 1	-3.33	0.44	*5.06 E-9*	(31)
IRGM	Immunity related GTPase M	-2.63	0.48	*7.55 E-6*	(32)
RAB9B	Ras-associated protein 9B	-6.25	1.76	*2.43 E-9*	(33)
RPS6KB1	Ribosomal protein S6 kinase B1	-2.56	0.13	*1.37 E-10*	(34)
SIRT1	Sirtuin 1	-2.56	0.14	*2.93 E-10*	(35,36)

References

1. Obara, K., Sekito, T., Niimi, K., and Ohsumi, Y. (2008) The Atg18-Atg2 complex is recruited to autophagic membranes via phosphatidylinositol 3-phosphate and exerts an essential function. *J. Biol. Chem.* **283,** 23972-23980
2. Velikkakath, A. K., Nishimura, T., Oita, E., Ishihara, N., and Mizushima, N. (2012) Mammalian Atg2 proteins are essential for autophagosome formation and important for regulation of size and distribution of lipid droplets. *Mol. Biol. Cell* **23,** 896-909
3. Hirata, E., Ohya, Y., and Suzuki, K. (2017) Atg4 plays an important role in efficient expansion of autophagic isolation membranes by cleaving lipidated Atg8 in Saccharomyces cerevisiae. *PLoS. One.* **12,** e0181047
4. Funderburk, S. F., Wang, Q. J., and Yue, Z. (2010) The Beclin 1-VPS34 complex- at the crossroads of autophagy and beyond. *Trends Cell Biol.* **20,** 355-362
5. Kang, R., Zeh, H. J., Lotze, M. T., and Tang, D. (2011) The beclin 1 network regulates autophagy and apoptosis. *Cell Death Differ.* **18,** 571-580
6. He, C., Wei, Y., Sun, K., Li, B., Dong, X., Zou, Z., Liu, Y., Kinch, L. N., Khan, S., Sinha, S., Xavier, R. J., Grishin, N. V., Xiao, G., Eskelinen, E. L., Scherer, P. E., Whistler, J. L., and Levine, B. (2013) Beclin 2 functions in autophagy, degradation of G protein-coupled receptors, and metabolism. *Cell* **154,** 1085-1099
7. Huang, C. C., Lee, C. C., Lin, H. H., and Chang, J. Y. (2016) Cathepsin S attenuates endosomal EGFR signalling: A mechanical rationale for the combination of cathepsin S and EGFR tyrosine kinase inhibitors. *Sci. Rep.* **6,** 29256
8. Crighton, D., Wilkinson, S., O'Prey, J., Syed, N., Smith, P., Harrison, P. R., Gasco, M., Garrone, O., Crook, T., and Ryan, K. M. (2006) DRAM, a p53-induced modulator of autophagy, is critical for apoptosis. *Cell* **126,** 121-134
9. Wang, Z., Miao, G., Xue, X., Guo, X., Yuan, C., Wang, Z., Zhang, G., Chen, Y., Feng, D., Hu, J., and Zhang, H. (2016) The Vici Syndrome Protein EPG5 Is a Rab7 Effector that Determines the Fusion Specificity of Autophagosomes with Late Endosomes/Lysosomes. *Mol. Cell* **63,** 781-795
10. Feng, Z. and Levine, A. J. (2010) The regulation of energy metabolism and the IGF-1/mTOR pathways by the p53 protein. *Trends Cell Biol* **20,** 427-434
11. Narendra, D., Tanaka, A., Suen, D. F., and Youle, R. J. (2008) Parkin is recruited selectively to impaired mitochondria and promotes their autophagy. *J. Cell Biol.* **183,** 795-803
12. Pallanck, L. (2013) Mitophagy: mitofusin recruits a mitochondrial killer. *Curr. Biol.* **23,** R570-R572
13. Munafo, D. B. and Colombo, M. I. (2002) Induction of autophagy causes dramatic changes in the subcellular distribution of GFP-Rab24. *Traffic.* **3,** 472-482
14. Neill, T., Torres, A., Buraschi, S., Owens, R. T., Hoek, J., Baffa, R., and Iozzo, R. V. (2014) Decorin induces mitophagy in breast carcinoma cells via peroxisome proliferator-activated receptor γ coactivator-1α (PGC-1α) and mitostatin. *J. Biol. Chem.* **289,** 4952-4968
15. Cerqua, C., Anesti, V., Pyakurel, A., Liu, D., Naon, D., Wiche, G., Baffa, R., Dimmer, K. S., and Scorrano, L. (2010) Trichoplein/mitostatin regulates endoplasmic reticulum-mitochondria juxtaposition. *EMBO Reports* **11,** 854-860
16. Fassan, M., D'Arca, D., Letko, J., Vecchione, A., Gardiman, M. P., McCue, P., Wildemore, B., Rugge, M., Shupp-Byrne, D., Gomella, L. G., Morrione, A., Iozzo, R. V., and Baffa, R. (2011) Mitostatin is down-regulated in human prostate cancer and suppresses the invasive phenotype of prostate cancer cells. *PLoS ONE* **6,** e19771
17. Vecchione, A., Fassan, M., Anesti, V., Morrione, A., Goldoni, S., Baldassarre, G., Byrne, D., D'Arca, D., Palazzo, J. P., Lloyd, J., Scorrano, L., Gomella, L. G., Iozzo, R. V., and Baffa, R. (2009) *MITOSTATIN*, a putative tumor suppressor on chromosome 12q24.1, is downregulated in human bladder and breast cancer. *Oncogene* **28,** 257-269
18. Choi, A. M. K., Ryter, S. W., and Levine, B. (2013) Autophagy in human health and disease. *New Engl. J. Med.* **368,** 651-662
19. Levine, B. and Kroemer, G. (2008) Autophagy in the pathogenesis of disease. *Cell* **132,** 27-42
20. Mizushima, N., Levine, B., Cuervo, A. M., and Klionsky, D. J. (2008) Autophagy fights disease through cellular self-digestion. *Nature* **451,** 1069-1075
21. Mizushima, N. and Levine, B. (2010) Autophagy in mammalian development and differentiation. *Nat. Cell Biol.* **12,** 823-830
22. Gewin, L., Zent, R., and Pozzi, A. (2017) Progression of chronic kidney disease: too much cellular talk causes damage. *Kidney Int.* **91,** 552-560
23. Pattingre, S., Tassa, A., Qu, X., Garuti, R., Liang, X. H., Mizushima, N., Packer, M., Schneider, M. D., and Levine, B. (2005) Bcl-2 antiapoptotic proteins inhibit Beclin 1-dependent autophagy. *Cell* **122,** 927-939
24. Pattingre, S. and Levine, B. (2006) Bcl-2 inhibition of autophagy: A new route to cancer? *Cancer Res.* **66,** 2885-2888

25. Espert, L., Denizot, M., Grimaldi, M., Robert-Hebmann, V., Gay, B., Varbanov, M., Codogno, P., and Biard-Piechaczyk, M. (2006) Autophagy is involved in T cell death after binding of HIV-1 envelope proteins to CXCR4. *J. Clin. Invest* **116,** 2161-2172
26. Hashimoto, I., Koizumi, K., Tatematsu, M., Minami, T., Cho, S., Takeno, N., Nakashima, A., Sakurai, H., Saito, S., Tsukada, K., and Saiki, I. (2008) Blocking on the CXCR4/mTOR signalling pathway induces the anti-metastatic properties and autophagic cell death in peritoneal disseminated gastric cancer cells. *Eur. J. Cancer* **44,** 1022-1029
27. Jung, C. H., Jun, C. B., Ro, S. H., Kim, Y. M., Otto, N. M., Cao, J., Kundu, M., and Kim, D. H. (2009) ULK-Atg13-FIP200 complexes mediate mTOR signaling to the autophagy machinery. *Mol. Biol Cell* **20,** 1992-2003
28. Yang, F., Zhang, W., Li, D., and Zhan, Q. (2013) Gadd45a suppresses tumor angiogenesis via inhibition of the mTOR/STAT3 protein pathway. *J. Biol. Chem.* **288,** 6552-6560
29. Zhang, D., Zhang, W., Li, D., Fu, M., Chen, R., and Zhan, Q. (2015) GADD45A inhibits autophagy by regulating the interaction between BECN1 and PIK3C3. *Autophagy.* **11,** 2247-2258
30. Bongers, K. S., Fox, D. K., Ebert, S. M., Kunkel, S. D., Dyle, M. C., Bullard, S. A., Dierdorff, J. M., and Adams, C. M. (2013) Skeletal muscle denervation causes skeletal muscle atrophy through a pathway that involves both Gadd45a and HDAC4. *Am. J. Physiol Endocrinol. Metab* **305,** E907-E915
31. Hu, B., Zhang, Y., Jia, L., Wu, H., Fan, C., Sun, Y., Ye, C., Liao, M., and Zhou, J. (2015) Binding of the pathogen receptor HSP90AA1 to avibirnavirus VP2 induces autophagy by inactivating the AKT-MTOR pathway. *Autophagy.* **11,** 503-515
32. Singh, S. B., Davis, A. S., Taylor, G. A., and Deretic, V. (2006) Human IRGM induces autophagy to eliminate intracellular mycobacteria. *Science* **313,** 1438-1441
33. Hirota, Y., Yamashita, S., Kurihara, Y., Jin, X., Aihara, M., Saigusa, T., Kang, D., and Kanki, T. (2015) Mitophagy is primarily due to alternative autophagy and requires the MAPK1 and MAPK14 signaling pathways. *Autophagy.* **11,** 332-343
34. Alers, S., Löffler, A. S., Wesselborg, S., and Stork, B. (2012) Role of AMPK-mTOR-Ulk1/2 in the regulation of autophagy: Crosstalk, shortcuts, and feedbacks. *Mol. Cell. Biol.* **32,** 2-11
35. Galluzzi, L., Kepp, O., and Kroemer, G. (2012) Mitochondria: master regulators of danger signalling. *Nat. Rev. Mol. Cell Biol.* **13,** 780-788
36. Kroemer, G., Marino, G., and Levine, B. (2010) Autophagy and the integrated stress response. *Mol. Cell* **40,** 280-293
